# Supplementary material for: Germline POT1 Deregulation Can Predispose to Myeloid Malignancies in Childhood
Source: Int J Mol Sci. 2021 Oct 26;22(21):11572. doi: 10.3390/ijms222111572 (PMC8583981; doi:10.3390/ijms222111572)
Supplement: Supplementary file 1 [file ijms-22-11572-s001.zip › Michler et al_Supplementary Figures_review_final.pptx]

## Slide 1
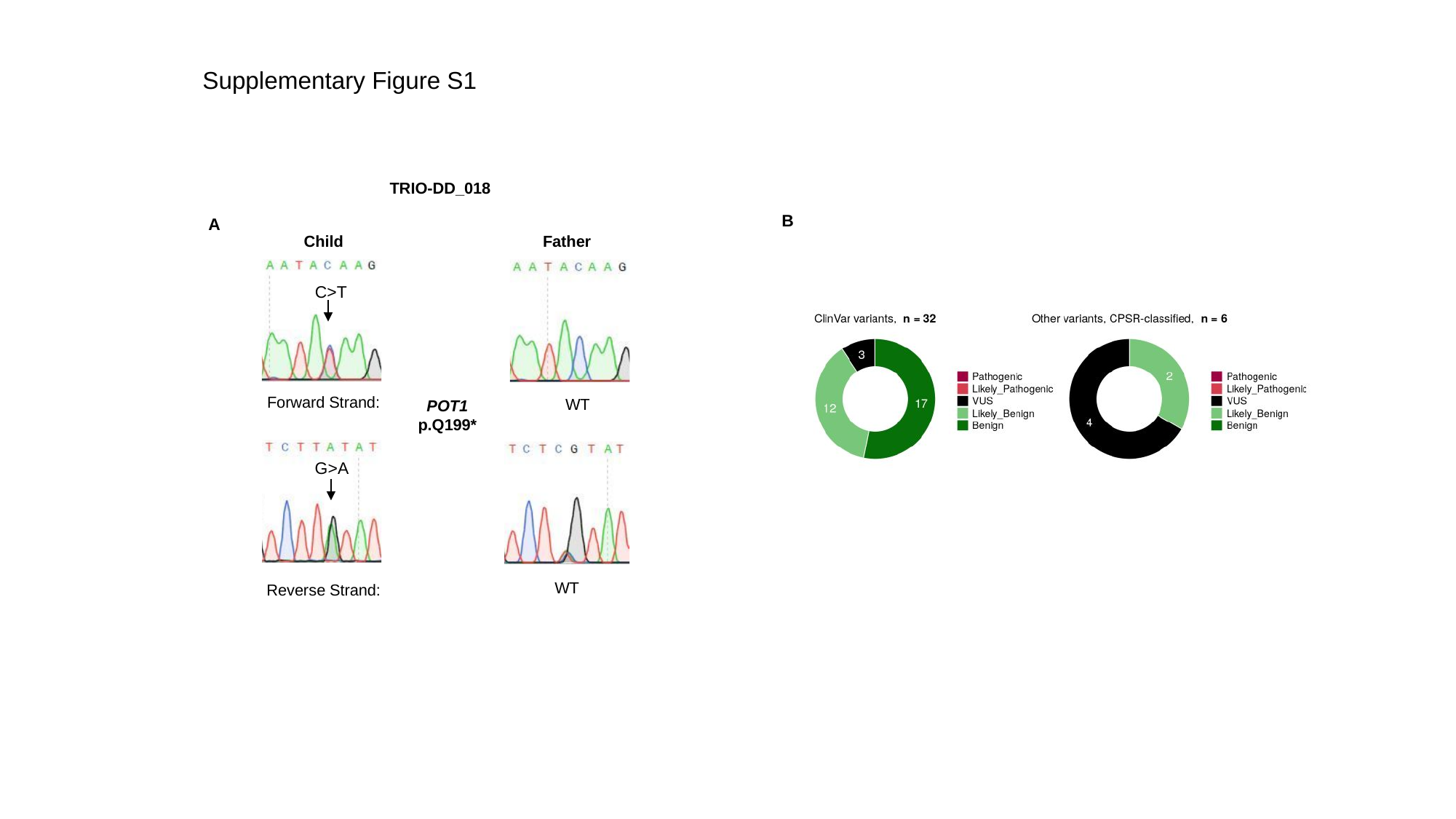

Supplementary Figure S1
TRIO-DD_018
B
A
Child
Father
C>T
Forward Strand:
WT
POT1
p.Q199*
G>A
WT
Reverse Strand:

## Slide 2
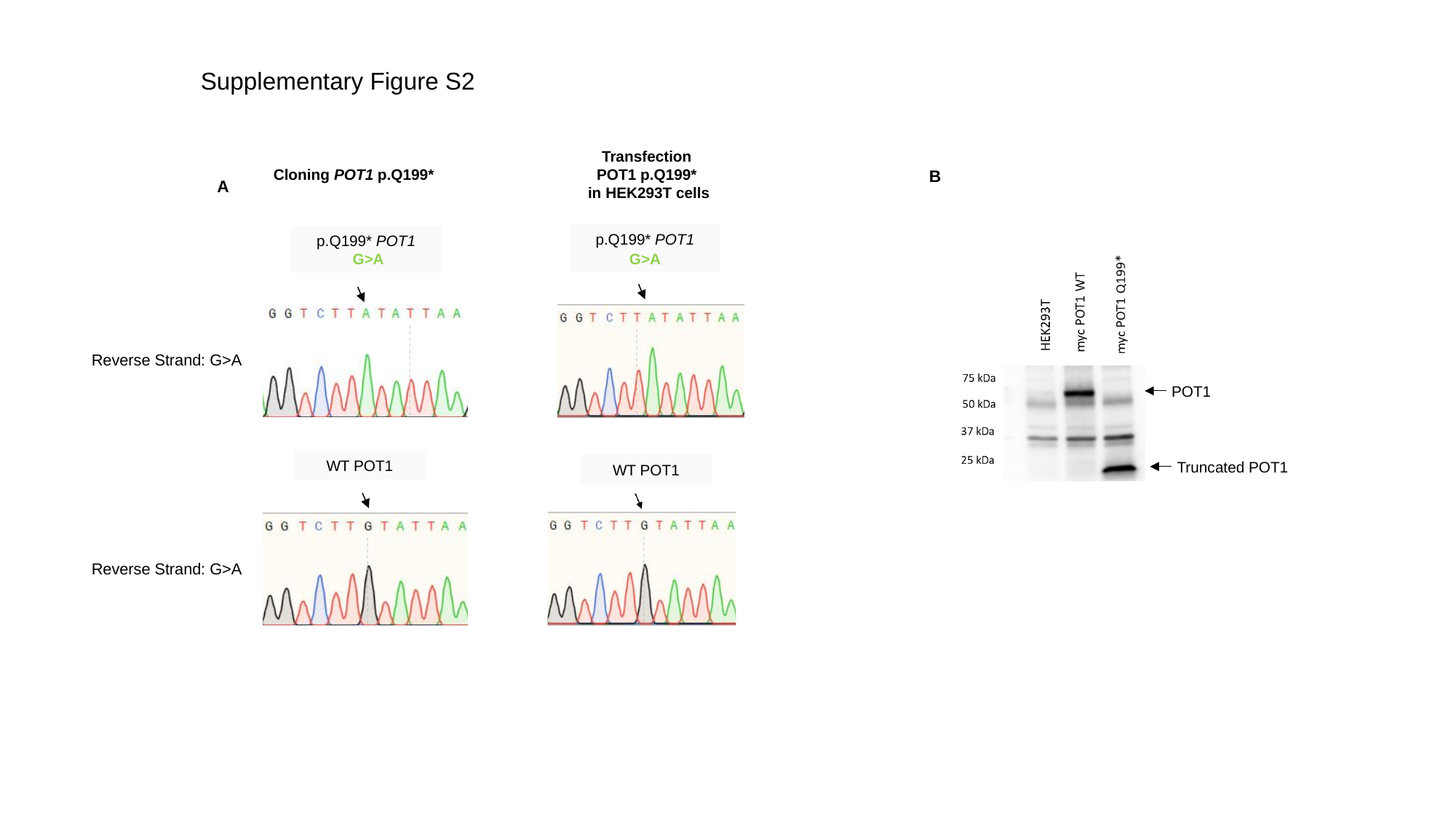

Supplementary Figure S2
Transfection
POT1 p.Q199*
in HEK293T cells
Cloning POT1 p.Q199*
B
A
p.Q199* POT1
G>A
p.Q199* POT1
 G>A
Reverse Strand: G>A
POT1
WT POT1
Truncated POT1
WT POT1
Reverse Strand: G>A

## Slide 3
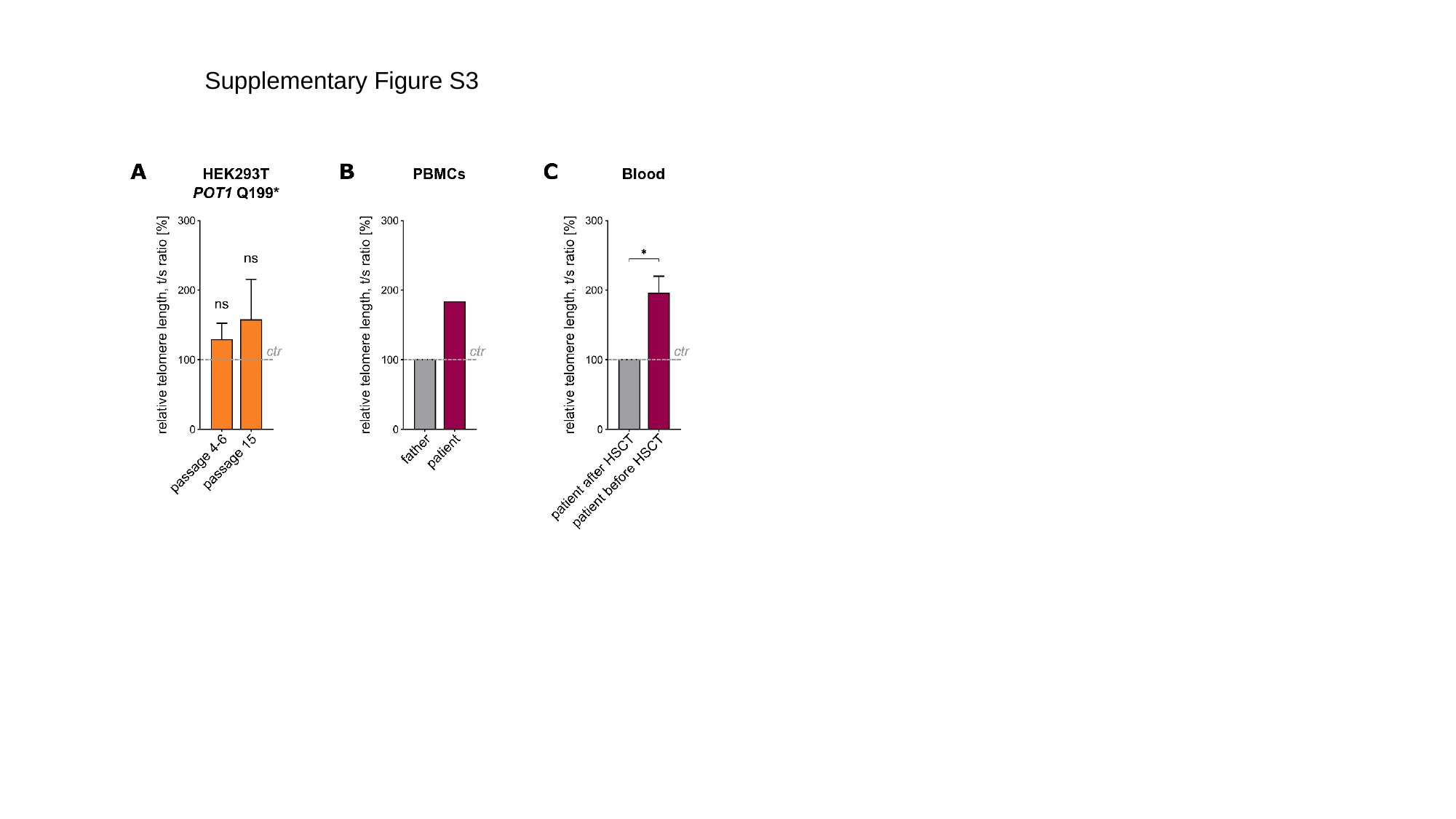

Supplementary Figure S3

## Slide 4
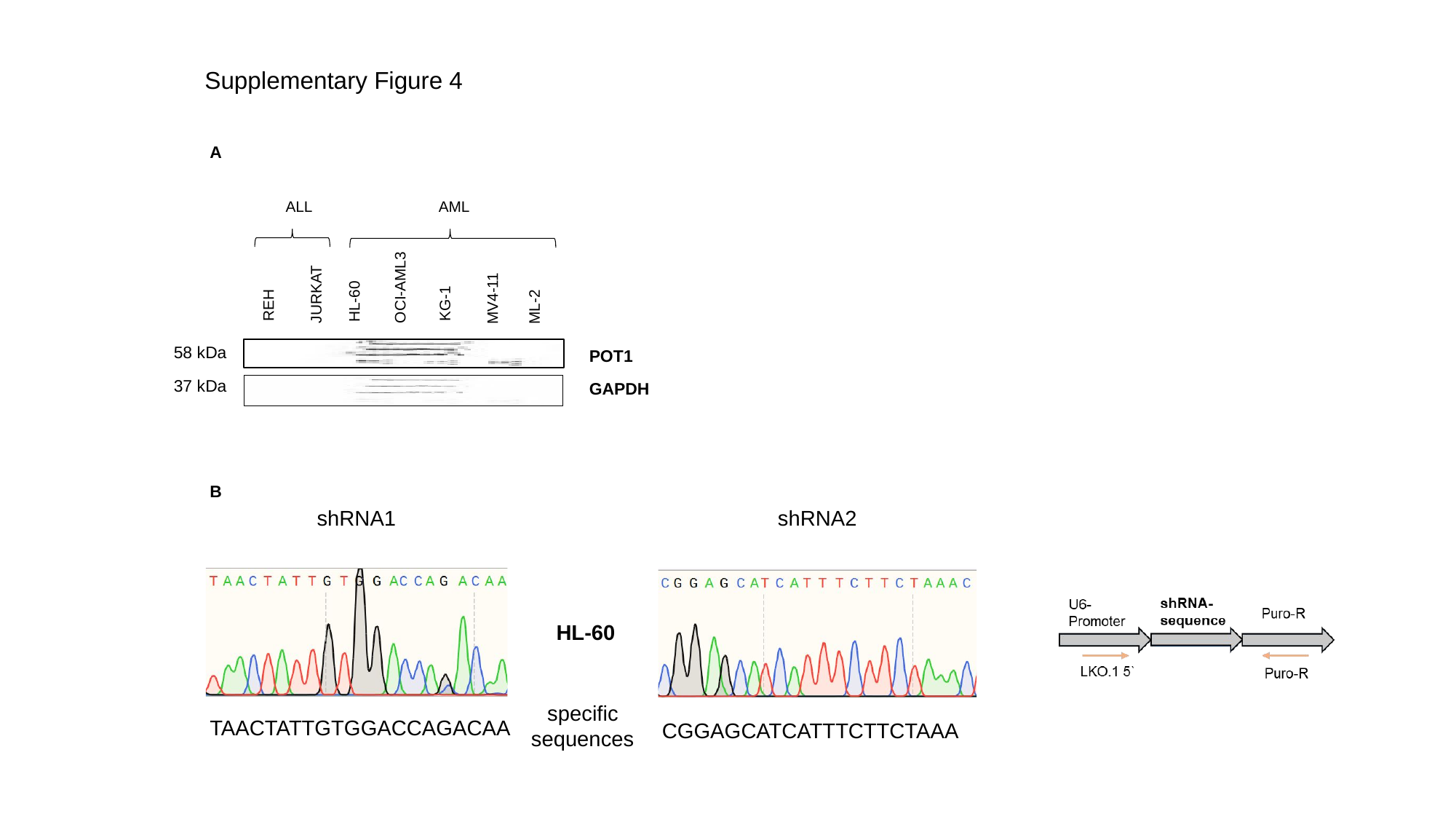

Supplementary Figure 4
A
ALL
AML
OCI-AML3
ML-2
JURKAT
MV4-11
KG-1
REH
HL-60
58 kDa
POT1
37 kDa
GAPDH
B
shRNA1
shRNA2
HL-60
specific sequences
TAACTATTGTGGACCAGACAA
CGGAGCATCATTTCTTCTAAA

## Slide 5
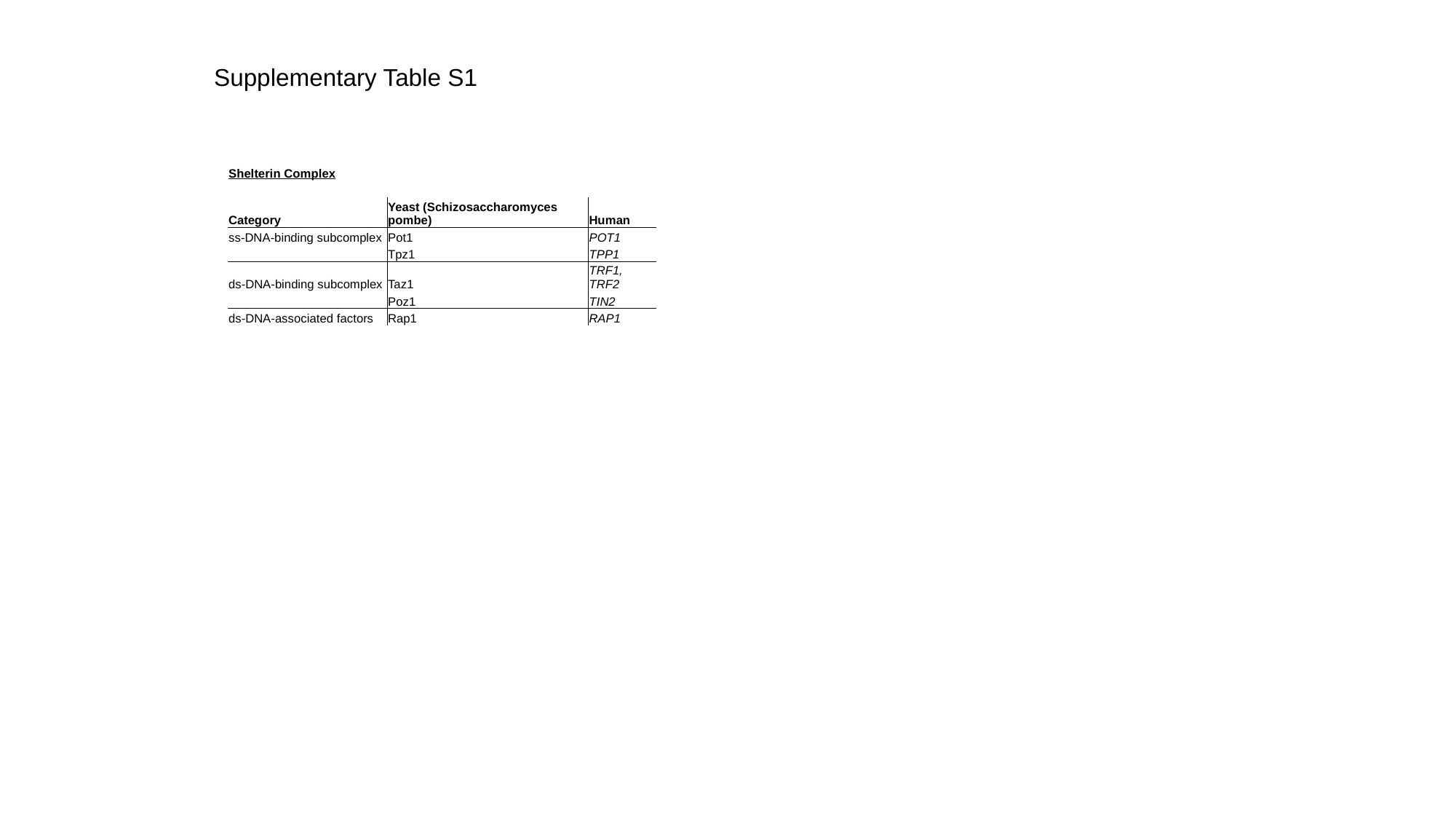

Supplementary Table S1
| Shelterin Complex | | |
| --- | --- | --- |
| | | |
| Category | Yeast (Schizosaccharomyces pombe) | Human |
| ss-DNA-binding subcomplex | Pot1 | POT1 |
| | Tpz1 | TPP1 |
| ds-DNA-binding subcomplex | Taz1 | TRF1, TRF2 |
| | Poz1 | TIN2 |
| ds-DNA-associated factors | Rap1 | RAP1 |

## Slide 6
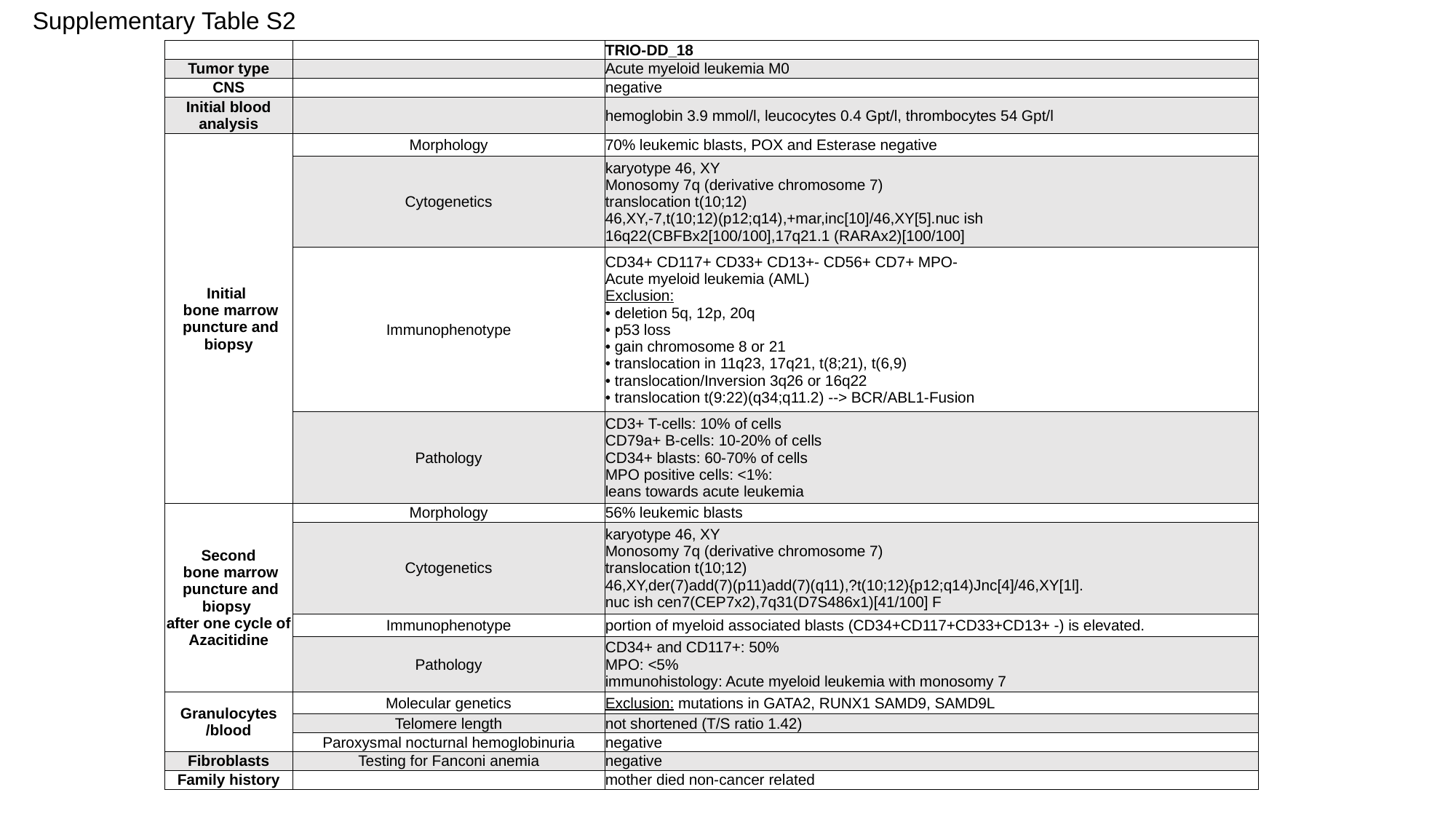

Supplementary Table S2
| | | TRIO-DD\_18 |
| --- | --- | --- |
| Tumor type | | Acute myeloid leukemia M0 |
| CNS | | negative |
| Initial blood analysis | | hemoglobin 3.9 mmol/l, leucocytes 0.4 Gpt/l, thrombocytes 54 Gpt/l |
| Initial bone marrow puncture and biopsy | Morphology | 70% leukemic blasts, POX and Esterase negative |
| | Cytogenetics | karyotype 46, XY Monosomy 7q (derivative chromosome 7) translocation t(10;12) 46,XY,-7,t(10;12)(p12;q14),+mar,inc[10]/46,XY[5].nuc ish 16q22(CBFBx2[100/100],17q21.1 (RARAx2)[100/100] |
| | Immunophenotype | CD34+ CD117+ CD33+ CD13+- CD56+ CD7+ MPO- Acute myeloid leukemia (AML) Exclusion: • deletion 5q, 12p, 20q • p53 loss • gain chromosome 8 or 21 • translocation in 11q23, 17q21, t(8;21), t(6,9) • translocation/Inversion 3q26 or 16q22 • translocation t(9:22)(q34;q11.2) --> BCR/ABL1-Fusion |
| | Pathology | CD3+ T-cells: 10% of cells CD79a+ B-cells: 10-20% of cells CD34+ blasts: 60-70% of cells MPO positive cells: <1%: leans towards acute leukemia |
| Second bone marrow puncture and biopsy after one cycle of Azacitidine | Morphology | 56% leukemic blasts |
| | Cytogenetics | karyotype 46, XY Monosomy 7q (derivative chromosome 7) translocation t(10;12) 46,XY,der(7)add(7)(p11)add(7)(q11),?t(10;12){p12;q14)Jnc[4]/46,XY[1l]. nuc ish cen7(CEP7x2),7q31(D7S486x1)[41/100] F |
| | Immunophenotype | portion of myeloid associated blasts (CD34+CD117+CD33+CD13+ -) is elevated. |
| | Pathology | CD34+ and CD117+: 50% MPO: <5% immunohistology: Acute myeloid leukemia with monosomy 7 |
| Granulocytes /blood | Molecular genetics | Exclusion: mutations in GATA2, RUNX1 SAMD9, SAMD9L |
| | Telomere length | not shortened (T/S ratio 1.42) |
| | Paroxysmal nocturnal hemoglobinuria | negative |
| Fibroblasts | Testing for Fanconi anemia | negative |
| Family history | | mother died non-cancer related |

## Slide 7
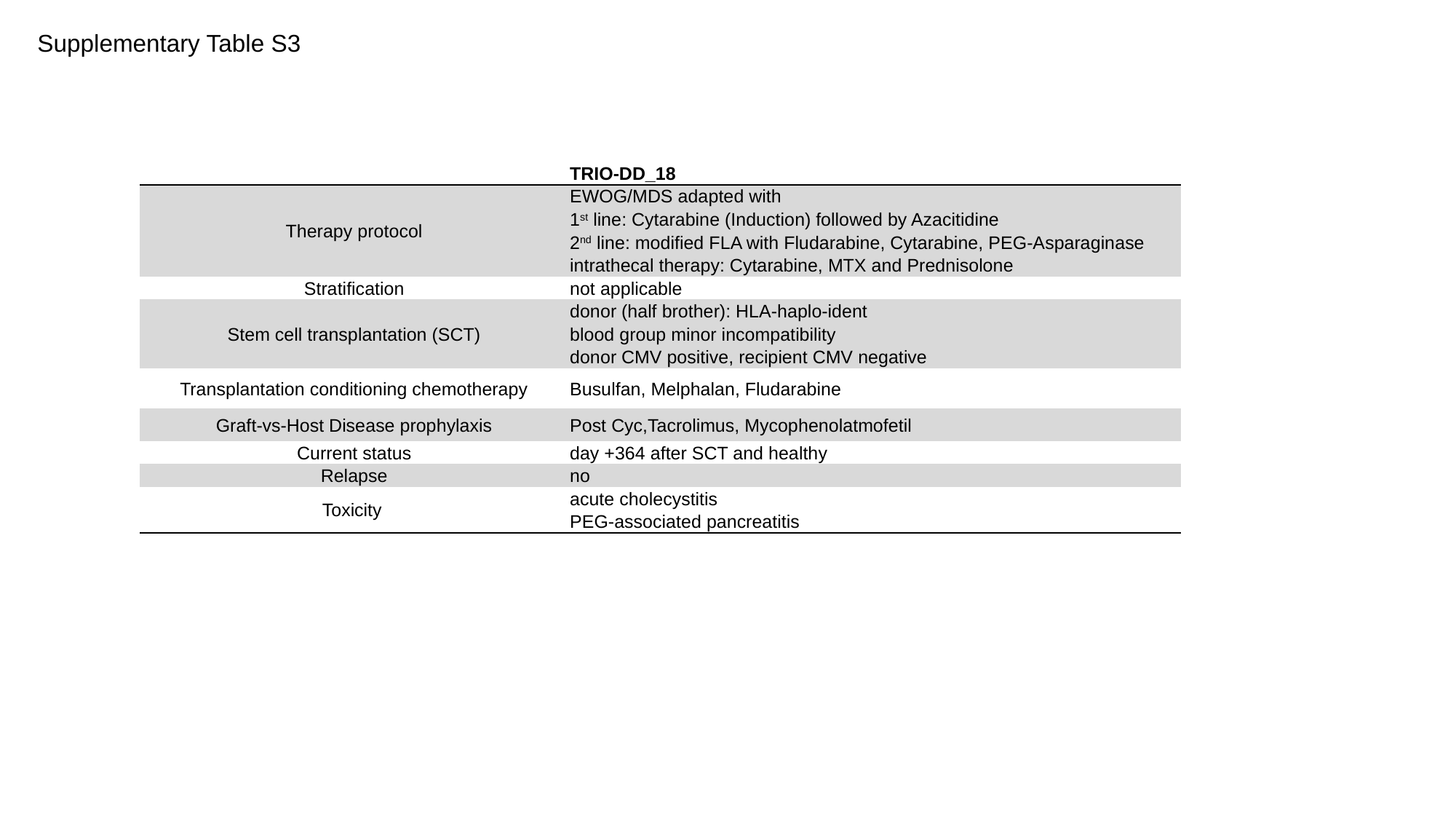

Supplementary Table S3
| | TRIO-DD\_18 |
| --- | --- |
| Therapy protocol | EWOG/MDS adapted with |
| | 1st line: Cytarabine (Induction) followed by Azacitidine |
| | 2nd line: modified FLA with Fludarabine, Cytarabine, PEG-Asparaginase |
| | intrathecal therapy: Cytarabine, MTX and Prednisolone |
| Stratification | not applicable |
| Stem cell transplantation (SCT) | donor (half brother): HLA-haplo-ident |
| | blood group minor incompatibility |
| | donor CMV positive, recipient CMV negative |
| Transplantation conditioning chemotherapy | Busulfan, Melphalan, Fludarabine |
| Graft-vs-Host Disease prophylaxis | Post Cyc,Tacrolimus, Mycophenolatmofetil |
| Current status | day +364 after SCT and healthy |
| Relapse | no |
| Toxicity | acute cholecystitis |
| | PEG-associated pancreatitis |
