# Supplementary material for: Germline POT1 Deregulation Can Predispose to Myeloid Malignancies in Childhood
Source: Int J Mol Sci. 2021 Oct 26;22(21):11572. doi: 10.3390/ijms222111572 (PMC8583981; doi:10.3390/ijms222111572)
Supplement: Supplementary file 1 [file ijms-22-11572-s001.zip › Supplementary Information_V2.pdf]

Supplementary Information (*Michler et. al.*)

Supplementary Figure Legends:

Supplementary Figure S1A: Genomic validation of *POT1* p.Q199*

Sanger-Sequencing for *POT1 p.Q199** variant confirmation was performed for TRIO-DD_018.

Supplementary Figure S1B: Analysis of pathogenic variants in patient carrying *POT1* p.Q199*

Analysis of TRIO-DD_018 variants by applying the Cancer Predisposition Sequencing Reporter (CPSR) algorithm. No pathogenic or likely pathogenic variants were found within the complete dataset.

Supplementary Figure S2A: Validation for cloning and transfection of POT1 p.Q199*

Sanger sequencing was performed for validation of cloning (DNA level) and transfection (mRNA level) of the *POT1* p.Q199* variant.

Supplementary Figure S2B: Western Blot analysis of transfected POT1 p.Q199* HEK293T cells

Western blot analysis of transfected HEK293T, WT and POT1 p.Q199* cells was performed as 3 independent experiments. Protein expression was visualized using a c-Myc antibody. We show that POT1 p.Q199* overexpression in HEK293T cells leads to a loss of around 2/3 of the WT protein.

Supplementary Figure S3: POT1 p.Q199* shows a trend for elongated telomeres

Relative telomere length (rTL) analysis by qRT-PCR (comparative ΔΔ-Ct method) with DNA isolated from transfected HEK293T cells (**A**), the patient's PBMCs compared to PBMCs from his father (**B**) and the patient's PBMCs before and after stem cell transplantation with cells from his haplo-identical half-brother (**C**). Figures displays 3 (HEK293T), 1 (PBMCs father) or 2 (PBMCs patient before and after transplant) biological replicates, respectively, each with 3 technical replicates, mean ±SEM. The data was normalized to the WT/father, which was set to 1. For the statistical analysis, the two-tailed Student's unpaired t-test was performed. HSCT = hematopoietic stem cell transplantation.

Supplementary Figure S4A: POT1 expression in ALL and AML cell lines

Western Blot analysis of POT1 expression in different ALL and AML cell lines. HL-60 as one of the AML cell line was selected for POT1 knockdown induction.

Supplementary Figure S4B: Validation of specific shRNA sequences in HL-60 POT1 knockdown cells

Sanger sequencing results of HL-60 POT1 knockdown cells. Specific shRNA1 and 2 sequences were amplified by PCR and successful integration was validated by Sanger sequencing.

Supplementary Table S1: Shelterin complex genes

Gene panel of the shelterin complex genes, adapted from *Moser* et al, 2010.

Supplementary Table S2: Presentation of leukemia in the index patient

Immuno- and molecular genetic tumor analyses performed for TRIO-DD_018.

Supplementary Table S3: Clinical characteristics of the index patient

Treatment history of TRIO-DD_018.
